# Evaluation on efficacy and safety of the addition of X-knife therapy to gefitinib in NSCLC patients with symptomatic brain metastases

**DOI:** 10.18632/oncotarget.10420

**Published:** 2016-07-06

**Authors:** Linbo Cai, Xiaoguang Qiu, Haihong Yang, Mingyao Lai, Changguo Shan, Weiping Hong, Juan Li, Longhui Luo, Ping Zhang, Lichao Wang

**Affiliations:** ^1^ Department of oncology, Guangdong 999 Brain Hospital, Guangzhou, China; ^2^ Department of neurosurgery of Beijing Tiantan Hospital, Capital Medical University, Beijing, China; ^3^ Department of oncology, the First Affiliated Hospital of Guangzhou Medical University, Guangzhou, China

**Keywords:** stereotactic radiosurgery, X-knife, EGFR, NSCLC, brain metastases

## Abstract

**Background:**

Stereotactic radiosurgery (SRS) is a widely used therapy for brain metastases(BMs) in Non-small cell lung cancer(NSCLC). However, its role in symptomatic patients with EGFR mutation remains unclear. We have retrospectively reviewed the clinical data of patients with symptomatic BMs whom received SRS as a salvage approach and concurrent gifitinib therapy.

**Methods:**

Seven patients with primary NSCLC, symptomatic BMs, and EGFR mutation were identified in a retrospective review of patients treated with SRS using X-knife at Guangdong 999 Brain Hospital between 1 January 2012 and 31 August 2014. The median follow-up of these patients was 16 months. Image fusion technique was used to determine cumulative doses to targeted lesions, whole brain, and critical brain structures. Toxicities and complications were identified by clinical records.

**Results:**

SRS(X-knife) was selected to be performed on seven patients (two males and five females) diagnosed with NSCLC and EGFR mutation due to the presence of encephaledema, compression of ventricles, or other complications. Neurological symptoms (such as paresis, aphasia, sensory and visual disturbances) were not present in any patients before or after SRS treatment, and the postoperative Karnofsky performance status(KPS) was improved in all patients. Median overall survival(OS) was 16 months and median progression free survival(PFS) was 10 months.

**Conclusions:**

The improvement of KPS and survival were reliable by SRS(X-knife) with concurrent gifitinib therapy in NSCLC patients with symptomatic BMs, and EGFR mutation. Given the small sample size, further prospective studies with a greater number of patients are warranted to confirm our results.

## INTRODUCTION

Non-small cell lung cancer(NSCLC) is the leading cause of cancer-related mortality worldwide. NSCLC patients are at high risk for brain metastases (BMs) with reported rates ranging from approximately 20% to 40% [1, 2]. Life expectancy for these patients is poor, with a median survival of only 3.4 months [3]. Moreover, many will suffer considerable loss of autonomy due to neurocognitive and functional deficits, as well as medication associated morbidity (such as steroid and antiepileptic drugs).

The outcomes of brain metastasis from NSCLC are both unfavorable and lacking in effective treatment options. Surgical resection is generally reserved for patients with a good prognosis, limited extracranial metastases, and a single brain lesion. Numerous trials have investigated chemotherapy in BMs patients, however, due to the inability of drugs to cross brain–blood barrier (BBB), there has been no categorically proven survival benefit [4, 5]. Whole brain radiotherapy (WBRT) has long been a mainstay of treatment for BMs but it is limited by long term side-effects. Recently, stereotactic radiosurgery (SRS) has become an alternative approach for patients with BMs; this procedure involves delivering a high dose of radiation, which allows for precise tumor targeting while minimizing the irradiation to the adjacent normal tissue [6, 7].

One previous meta-analysis demonstrated that adding SRS to WBRT improved survival in patients with one brain metastasis, and improved local tumor control and functional independence in all patients [8]. Nevertheless, three randomized trials consistently found that compared with SRS alone, the use of WBRT plus SRS did not improve survival for patients with BMs [9–11]. In addition, Xue et al. investigated the safety of radiotherapy and found that WBRT resulted in a higher incidence of radiation-related toxicities than SRS [12]. Overall, evidence is in favor of SRS as an effective therapy in BMs patients despite differences in patient selection and treatment design.

Several reports have shown that the special tyrosine kinase inhibitor (TKI) of the epidermal growth factor receptor (EGFR), is capable of reducing BMs in NSCLC, sometimes with a highly dramatic response [13–15]. The status of EGFR mutation has been reported to be associated not only with improved survival for patients with BMs [16], but also with the response rate of radiotherapy [17]. However, a recent prospective study showed the addition of TKI to WBRT+SRS in NSCLC patients with 1 to 3 brain metastases did not improve survival and may even have had a deleterious effect [18]. The contradictory results of this study and the others, detailed above, involving WBRT in combination with SRS raise the question of whether TKI alone should be the primary treatment option, with SRS reserved only as a salvage method in patients with symptomatic BMs. Herein, We report the results of the addition of SRS (X-knife) to TKI (gefitinib) therapy in EGFR mutated NSCLC patients with symptomatic BMs to evaluate its’ safety and efficacy.

## MATERIALS AND METHODS

### Patient characteristics

We retrospectively retrieved the medical records of seven consecutive patients who were diagnosed as NSCLC with severe symptomatic BMs at Guangdong 999 Brain Hospital between 1 January 2012 and 31 August 2014. This study was reviewed and approved by the ethics committee. Included patients must have had pathologically confirmed NSCLC with EGFR gene mutation, and medical image confirmed brain metastasis. Conventional therapy included gefitinib, with SRS(X-knife) reserved as the salvage method. Contrast-enhanced CT and positron emission tomography CT (PET-CT) were performed for evaluation. Staging was designated by the 7^th^ edition of the tumor, node, metastasis (TNM) classification.

### Samples and EGFR mutation detection

All patients had adequate tumor tissue or biopsy samples for molecular analysis. The EGFR Mutation Detection Kit (Amoy Diagnostics, Xiamen, China), which is based on the amplification mutation refractory system(AMRS) technology, was used to detect the 29 most common types of EGFR mutations and the T790M mutation. All experiments were performed according to the user manual. Tissues came from operations in three patients (one from the lung, two from the head), and biopsy of the lung in the other four. Though not all samples were from the BMs, a previous Chinese study identified an accordance rate of 93.3% in the EGFR mutation status between the primary tumor and BMs [19]. Hence, primary tumor EGFR status is a very good surrogate for EGFR mutation status of the BMs.

### Treatment procedures

EGFR TKI (Gefitinib) was used as the standard treatment as soon as the mutation was diagnosed. X-knife image fusion software (Radionics RT2, Burlington, MA) was used to fuse all treatment images and data to a reference CT image (initial CT before the first SRS treatment). Cumulative and individual doses to each lesion, the whole brain, the optic chasm, and the right and left optic nerves were determined for each patient with the X-knife planning software. The system included a 6-MV dedicated linac with fixed circular cones (Varian 600C/D); this was used with the X-knife forward-planning system.

### Follow-up evaluations and primary outcomes

Changes in the neurological symptoms, such as paresis, aphasia, sensory and visual disturbances, were examined before and after treatment in all patients. The severity of symptoms was divided into four grades based on the activities of daily living determined according to the medical care accreditation criteria: Grade 0: no trouble (able to perform the activities without help), Grade 1: slightly impaired (able to perform/complete? activities with some difficulty), Grade 2: moderately affected (needing partial support), and Grade 3: severely affected (unable to function in normal daily life and needing total support). In addition, the improvement of symptoms was defined as an increase by one grade or more.

A Magnetic Resonance Imaging(MRI) or CT-scan were performed before treatment and every one to four months thereafter, to define the tumor response and local control. The primary end point of the study was overall survival (OS) calculated from the start of gefitinib treatment to the date of death. Secondary end points included progression-free survival (PFS) defined as the time elapsed between gefitinib treatment and disease progression. The activity of daily living was assessed using Karnofsky performance status(KPS) index.

## RESULTS

### Patient characteristics and radiologic and pathologic findings

A total of seven patients’ (two males and five females) clinical data were analyzed in the present study. Patient age ranged from 44 to 62. All patients were pathologically diagnosed as adenocarcinoma according to the 7^th^ lung cancer classification. In regard to EGFR mutation, five patients had 19 deletion and two had 20 deletion. No T790M mutation was found. Among the included patients, five had more than four BMs, one had one BM (size of 6.0*3.0cm), and one had widely reinforced in pia mater. Preoperative KPS score ranged from 30 to 90. The details are listed in Table 1.

**Table 1:** Patient characteristics and radiologic and pathologic findings

Patient ID	Gender	Age	Smoke	Region of specimen	EGFR mutation	Neurological manifestation (Grade0,1,2,3)	Number of lesions in brain (the size of the biggest one)	Location of lesions
**1**	Female	59	No	Biopsy	19 del+	3	Widely reinforcement in pia mater (2.0*1.5cm)	Pia mater
**2**	Male	44	No	Biopsy	19 del+	2	6 (3.7*3.3cm)	Bilateral frontal lobe, parietal lobe on the right side, and four ventricle on the rim
**3**	Female	44	No	Biopsy	19 del+	2	6 (5.2*3.1cm)	Bilateral frontal occipital lobe
**4**	Male	58	No	Surgery	20 del+	0	12 (3.0*2.5cm)	Bilateral cerebral hemisphere, cerebellar hemisphere and the left cerebral peduncle
**5**	Female	50	No	Biopsy	19 del+	2	4 (5.2*4.1cm)	Bilateral frontal, temporal, parietal lobe, left occipital lobe, cerebellum awning and bilateral cerebral hemisphere pia mater
**6**	Female	62	No	Surgery	19 del+	0	1 (6.0*3.0cm)	Right parietal, occipital lobe,
**7**	Female	53	No	Surgery	20 del+	2	20 (1.29*1.22cm)	Bilateral cerebral hemisphere, cerebellar vermis, bilateral basal ganglia region

### Treatment efficacy and safety

**Figure 1:**
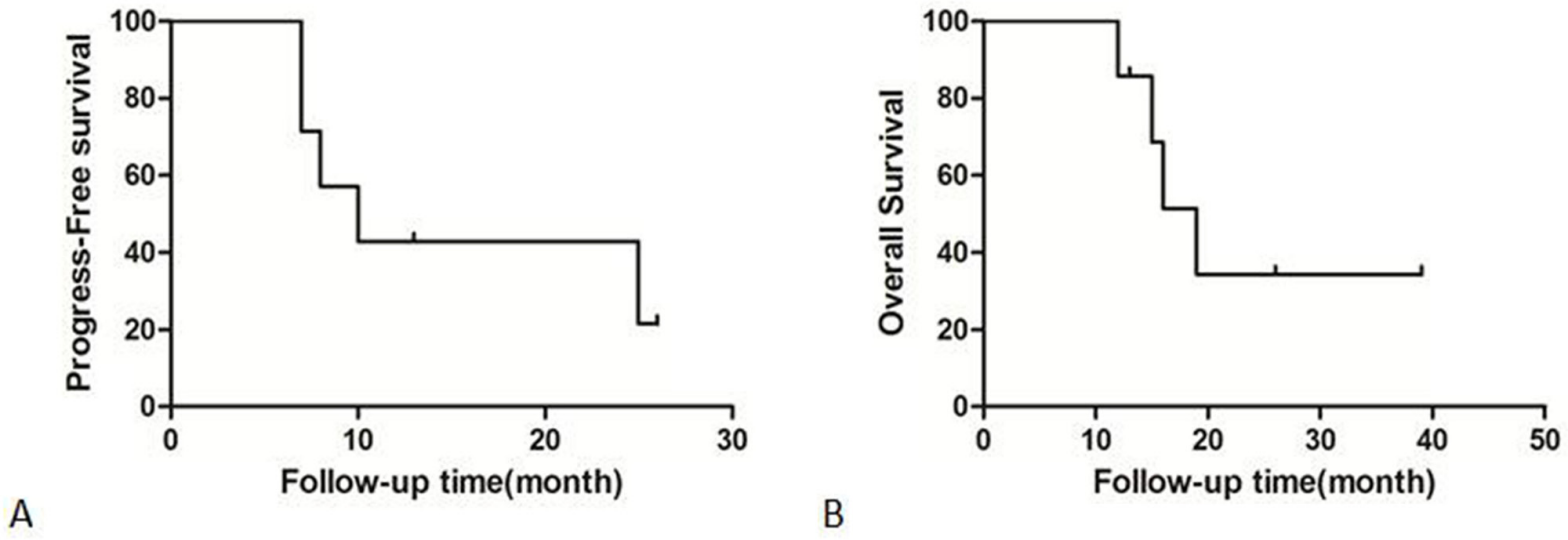
**A.** The progress-free survival of included patients. **B.** The overall survival of included patients.

In the present study, two of the included patients received gefitinib as second line treatment. Two of the patient received the surgery for brain metastasis first, then received SRS, then received gefitinib. The others received biopsy first, then received gefitinib, then received SRS. Duration of Gefitinib usage ranged from two to thirteen months. X-knife was performed in all patients. Steroids were used after radio-surgery and stopped within one week. Median overall survival was 16 months, and median PFS was 10 months (Figure 1). Patients were found to tolerate treatment well. The KPS score ranged from 70 to 90 one week after X-knife therapy. Demographic details, and radio-surgical data for all patients are summarized in Table 2.

**Table 2:** Treatment efficacy and safety

Patient ID	Reason to use SRS	SRS treatment strategies	Usage of corticosteroid after SRS treatment	Duration of gefitinib treatment	Neurological manifestation after SRS (Grade0,1,2,3)	KPS score before SRS treatment	KPS score after SRS treatment	PFS (months)	OS (months)
**1**	Brain edema	One isocentre with 1000cgy dose on one lesion	Stop at seven days after SRS	9	1	30	60	7	12
**2**	Ventrcles compression	First time: one isocentre with 1000cgy dose on one lesion; Second time: five isocentres with 1200cgy on four lesions	Stop at five days after SRS	2	0	60	90	8	19
**3**	Brain edema	Seven isocentres with 1300cgy on six lesions	Stop at seven days after SRS	6	1	70	80	7	15
**4**	Brain edema	First time: nine isocentres with 1000-1400cgy on nine lesions; Second time: nine isocentres with 1000-1400cgy on nine lesions	Stop at seven days after SRS	10	1	80	80	10	16
**5**	Brain edema	Eight isocentres with 1400cgy on eight lesions	Stop at five days after SRS	12	1	60	90	26+	26+
**6**	Ventrcles compression	One isocentre with 1000cgy dose on one lesion	Stop at five days after SRS	13	1	90	90	13+	13+
**7**	Brain edema	Nine isocentres with 1000-1400cgy on nine lesions	Stop at seven days after SRS	12	2	70	80	25	39+

## DISCUSSION

BMs is a common and lethal complication of NSCLC, which portends a poor prognosis. Radiotherapy has been applied to inoperable BMs as a therapeutic or palliative option. The use of drugs targeting the proteins of mutated EGFR has become a standard of care in the systemic treatment of metastatic NSCLC as well [20]. While the simultaneous presence of BMs and EGFR mutations are common , the efficacy and safety of the additional use of SRS to TKI in symptomatic NSCLC patients remains unclear. In the present study, we demonstrated that the functional autonomy (KPS) and survival could be improved by SRS(X-knife) as a salvage approach in symptomatic BMs patients.

**Figure 2:**
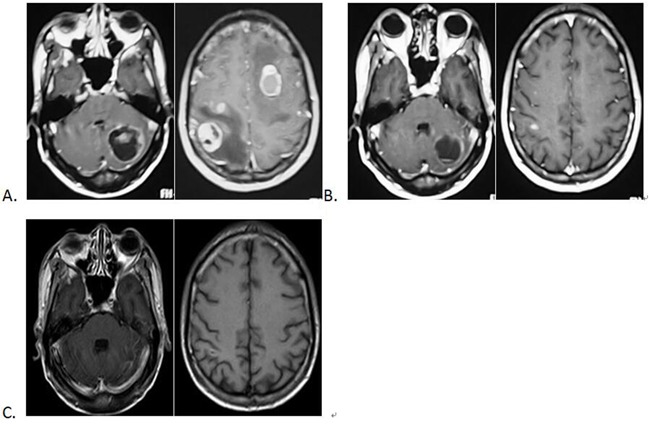
MRI images showing brain metastases of NSCLC **A.** Before the SRS treatment; **B.** One month after SRS treatment; **C.** Four months after SRS treatment.

SRS delivers a single high dose of radiation to the target volume while avoiding the surrounding normal tissues. Hall and colleagues reported that BMs patients treated with SRS alone have similar overall survival and receive more cost-effective care than those treated with SRS+WBRT [21]. Another study by Soffietti et al. concluded patients who received WBRT experienced worse health related quality of life (HRQOL), particularly during the early follow-up period, compared with patients treated with surgery or SRS alone [22]. As a result, SRS has been confirmed to be an effective and less invasive therapy. In the present study, SRS was performed as a salvage therapy for patients with complications, including encephaledema and compression of ventricles, and all patients were diagnosed with lung adenocarcinoma and severe BMs,. In addition, all included patients received EGFR TKI before SRS and the total radiation doses received were well tolerated. After SRS treatment, the KPS scores of all patients increased. Postoperative MRI examination also revealed either stable or smaller tumor lesions (Figure 2).

EGFR mutation status is associated with improved survival among patients with BMs [16]. The study from Cai et al. reported that administration of TKI agents with WBRT/SRS/surgery might be beneficial for OS and PFS of intracranial disease and PFS of extracranial disease in NSCLC patients with BMs independent of EGFR mutations [23]. However, Sperduto and colleagues found that the addition of TKI to WBRT+SRS in NSCLC patients with 1 to 3 brain metastases did not improve survival and possibly had a deleterious effect [18]. In our study, the seven included patients had primary tumor tissues or BMs diagnosed, EGFR mutation(5, 19 deletion/2, 20 deletion) and received gefitinib as primary treatment. The median OS was 16 months and the median PFS was 10 months.

In general, BMs are resistant to systemic chemotherapy due to the inability of drugs to cross brain–blood barrier (BBB) [4, 5]. Recently, the remarkable response and mild toxicity of EGFR TKI treatment makes it an attractive option for NSCLC patients with BMs and EGFR mutations [24]. One prospective study of Gefitinib on NSCLC patients with BMs reported that the response rate (RR) of TKI use in BMs patients was 10%, with a median duration of response of 13.5 months and median OS of 5 months [15]. However, another study consisting of 15 patients reported that the RR of TKI use in BMs was 60%, with a median duration of response of 8.7 months [25]. The increased RR is most likely attributed to previous radiation therapy. In addition, erlotinib seems to produce higher CSF levels than gefinitib [26], and therefore might be preferable. Another limitation is that the EGFR mutation detection kit we used can only detect three kinds of EGFR exon 20 insertion.

In conclusion, SRS(X-knife) with concurrent gifitinib therapy in NSCLC patients with symptomatic BMs, and EGFR mutation produced reliable improvement of KPS and survival. Given the small sample size, further prospective studies with a greater number of patients are warranted to confirm our results.
